# The Microscope in Medicine

**Published:** 1871-04

**Authors:** 


### Selections.

### THE MICROSCOPE IN MEDICINE.

The debt which modern medicine, both theoretical and practical, owes to the microscope can hardly be overestimated, and yet the value of the instrument for clinical purposes is not so generally appreciated by physicians as it ought to be. Many are too ready to sneer at it as a mere plaything, and to deny that it is of real service to the every-day work of a medical man. To such skeptics we commend the following extracts from a chapter on the microscope in a recent work by Prof. Bennett of the University of Edinburgh:—

"Some years ago I was summoned to see a dispensary patient laboring under bronchitis, who was spitting florid blood. On examining the sputum with a microscope, I found that the colored blood corpuscles were those of a bird. On my telling her she had mixed a bird's blood with the expectoration, her astonishment was unbounded, and she confessed that she had done so for the purpose of imposition.

"A boy was brought to me with an eruption on the scalp, which was of so indefinite a character that its nature could not be determined. He had lately been elected to occupy a vacancy in one of our educational charitable establishments, and the question to decide was, whether the disease was or was not contagious. On examining the scalp with a microscope, I readily discovered the *Achorion Schoenlenci*, or fungus constituting true favus, and as this has been experimentally proved to be inoculable, I had no hesitation in preventing his admission into the school.

"A child was brought to me said to be affected with worms, because it passed yellow shreds in abundance, which, to the naked eye, closely resembled ascarides. All kinds of vermifuge remedies had been tried in vain. On examining the shreds with a microscope, I found them to consist of undigested spinal vessels of plants, and they ceased to appear when the vegetable broth used as food was abandoned.

"An individual was supposed to be laboring under dysentery, from the frequent passage of yellowish pulpy masses in the stools, accompanied with tormina and other symptoms. On examining these masses with the microscope, I found them to consist of undigested potato skins. On inquiry it was found that this person had eaten the skins with the potatoes. On causing these to be removed before dinner, the alarming appearance ceased, and the other symptoms also disappeared.

"An elderly lady conceived herself to be affected with insects continually forming on the skin, which produced incessant itching and tingling. All the hair was removed, and every kind of application was tried without effect. On rubbing the surface she always saw minute white rolls and black specks, which she regarded as insects in different stages of development. The torment and anxiety this caused her for many months it is scarcely possible to conceive. At length she labored under the idea that she was communicating the disease to her husband and daughter, when, at the request of her medical attendant in the west of Scotland, she came to Edinburgh in order that I might investigate and treat it. I had the pleasure of showing this lady, under the microscope, that the white bodies were minute rolls of epidermis or of the cotton cloth with which she rubbed the skin, and that the black specks were portions of dust or soot. Her hallucination being in this way dissipated, she returned home perfectly well."—*Boston Journal of Chemistry.*

